# Curcumin-Loaded High-Charge Swelling Synthetic Mica: Characterization Studies and Stability under Stress Conditions

**DOI:** 10.1021/acs.langmuir.5c01163

**Published:** 2025-05-23

**Authors:** María del Mar Orta Cuevas, Ana Fernández Romero, Antonio M. Rabasco Álvarez, Santiago Medina-Carrasco, María Luisa González-Rodríguez

**Affiliations:** † Department of Analytical Chemistry, Faculty of Pharmacy, 16778 Universidad de Sevilla, C/Profesor García, González 2, 41012 Sevilla, Spain; ‡ Department of Pharmacy and Pharmaceutical Technology, Faculty of Pharmacy, 16778 Universidad de Sevilla, C/Profesor García, González 2, 41012 Sevilla, Spain; § X-ray Laboratory, CITIUS, 16778 Universidad de Sevilla, Avenida Reina Mercedes, 4B, 41012 Sevilla, Spain

## Abstract

Curcumin (Cur) is a bioactive compound with various pharmacotherapeutic effects. However, its limited solubility and stability pose challenges for therapeutic applications. Clay minerals, such as montmorillonite (MMT) and high-charge swelling synthetic micas, show promise as drug carriers due to their properties. This study aimed to obtain complexes with clay minerals that could enhance the stability of Cur. MMT, Na-Mica-4, and the latter organofunctionalized with 18-carbon alkylamines (C18-Mica-4) were used as support for Cur. Adsorption studies showed that Na-Mica-4 exhibited the highest percentage of adsorption (60%). Cur-Mica-4 complexes were characterized by X-ray diffraction (XRD), Fourier transform infrared spectroscopy (FTIR), thermal analysis (DSC and TGA), specific surface area (BET), pore size and volume determinations, and surface charge determination by zeta potential measurement. The effect of light on Cur and Cur-Mica-4 complexes was also evaluated. Forced degradation studies were performed under hydrolytic, oxidative, photolytic, and thermal conditions to assess the stability and degradation pathways. The FTIR spectra indicated that the enol tautomer mainly formed part of the complexes. BET analysis showed a reduced pore size after adsorption, indicating Cur immobilization. TGA and scanning electron microscopy (SEM) suggested degradation primarily occurring under exposure to sunlight, heat, and ultraviolet light. The effect of acidic and basic conditions on the Cur-Mica-4 complex was evaluated. Under acidic conditions, a decrease in the specific surface area of the complex was observed, suggesting the formation of larger configurational structures. An increase in the specific surface area with a smaller pore size was observed in the basic medium, possibly due to the formation of new structures in the clay minerals, supported by XRD results. These findings indicate that the pH of the medium can significantly influence the structure and stability of the Cur-Mica-4 complex, which could have important implications for its application in specific environments, such as drug delivery systems.

## Graphical abstract

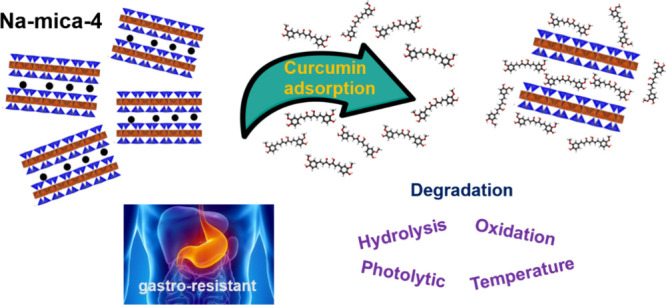

## 1 Introduction

Curcumin (Cur), a yellow pigment known chemically as diferuloylmethane, is a class IV BCS drug and a natural polyphenolic bioactive ingredient obtained from the rhizome isolate of Curcuma longa L.^1,2^ The chemical structure is based on [(*E*,*E*)-1,7-bis­(4-hydroxy-3-methoxyphenyl)-1,6-heptadiene-3,5-dione] ^3,4^ consisting of two rings with phenolic OH groups linked by an unsaturated diketone (Figure 1).

**Figure 1:**
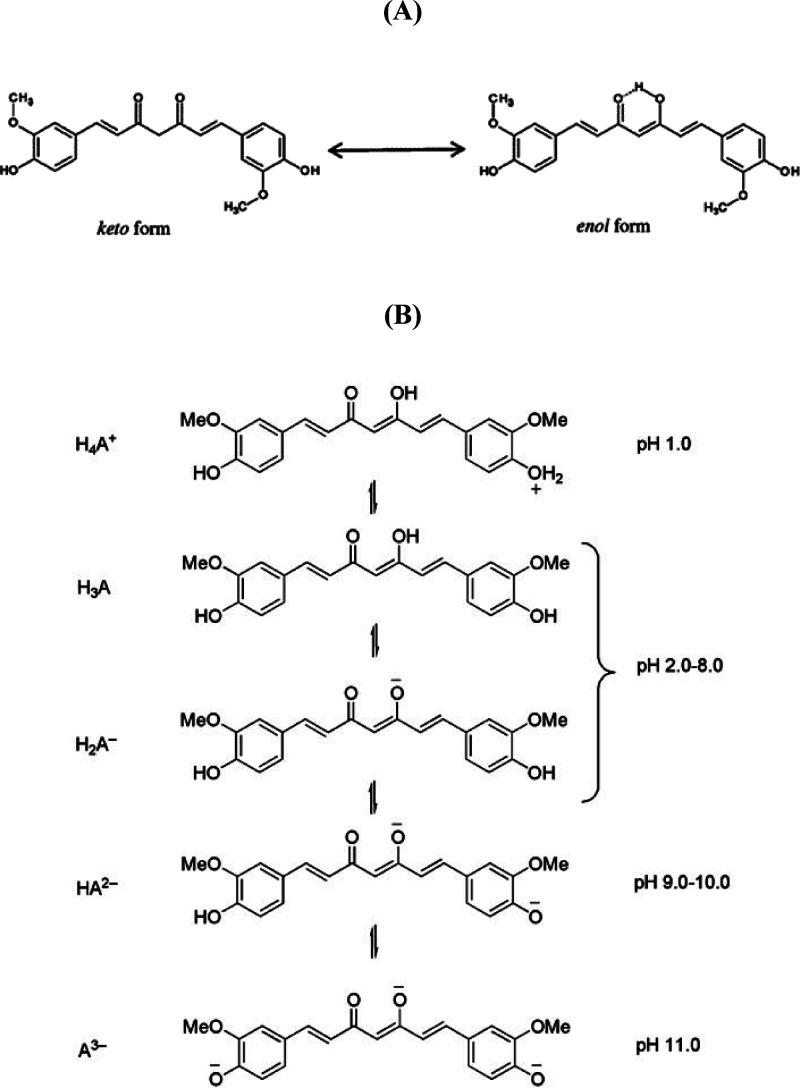
(A) Chemical structure of curcumin (Cur) and the coexisting forms keto and enol. (B) Acid–base equilibrium of Cur.

The solubility and stability of Cur are closely related to its chemical structure. It has three reactive functional entities in its structure that involves two aromatic ring systems containing *o*-methoxy phenolic groups, connected by a seven-carbon linker consisting of an α,β-unsaturated β-diketone moiety.^5^ The diketone structure undergoes keto–enol tautomerism depending on the environment,^6^ as shown in Figure 1A. It is practically insoluble in water at a neutral and acidic pH; however, solubility increases in the presence of organic solvents, such as ethanol. In aqueous solutions and at alkaline pH, the acid phenol group in Cur dissociates its hydrogen, forming the phenolate ion that makes the solubility of Cur in water possible (Figure 1B). In acidic and neutral solutions, the β-diketo form is prevalent, whereas in alkaline environments, the keto–enol form predominates, associated with Cur.^4,7^ In addition, the enol form is more chemically labile than the keto form, contributing to the poor chemical stability of Cur in basic solutions.

However, ketone groups are involved in the colorimetric properties of Cur. In 2021, Prasad et al. managed to isolate the two tautomers and detect the presence or absence of ketone groups by NMR.^8^

In recent years, Cur has been investigated for a wide range of applications in medical sciences due to its pharmacotherapeutic effect as antioxidant, anti-inflammatory, anticancer, antihepatotoxic, antidepressant, antibacterial, antifungal, antimalarial, antiviral, or antidiabetic properties.^3,4,9–11^ Despite interest in the medical field, its therapeutic use is limited due to the drawbacks of its properties, such as poor solubility in neutral and acidic media, being practically insoluble in water and with low systemic concentrations after oral administration, as the gastrointestinal tract (basic conditions) is unstable.^12,13^ Furthermore, it has a short half-life and low bioavailability after topical administration due to its lipophilicity and low permeability.^1,9,14,15^ In pharmaceutical and biomedical fields, clay minerals are being investigated as biocompatible and nontoxic nanomaterials that act as carriers of active ingredients due to their properties, such as natural abundance, large surface area, low cost, swelling capacity, high adsorption capacity, chemical stability, reactive functional groups, and surface charge. This strategy would control the stability and increase drug loading, improve drug safety through controlled and targeted delivery, and improve low solubility and bioavailability properties by increasing the dissolution rate.^16–19^ Although the use of clays in pharmaceutical formulation is advantageous in general, there are some risks associated with prolonged administration of clay minerals. The long-term oral administration caused kidney stone formation and the adsorption of some nutritive elements and enzymes, leading to their elimination from the body.^16^ In the literature, there are very few studies on Cur adsorption on clays, which reinforces the novelty of this work. In this study, clay minerals used as Cur nanocarriers were montmorillonite (MMT), a synthetic highly expansible mica (Na-Mica-4) and Na-Mica-4 organically functionalized with cations of octadecylamine (C18-Mica-4).^16,18,20^ MMT is a 2:1 dioctahedral phyllosilicate with the formula (Na,Ca)_0.33_(Al,Mg)_2_(Si_4_O_10_)­(OH)_2_·nH_2_O), consisting of an octahedral alumina layer surrounded by two opposing tetrahedral silica layers, belonging to the smectite group.^21^ The induced charge within the layers by isomorphic cation substitution leads to the formation of an electrostatically charged structure, and the negative charges of the layers are compensated by these cations. This feature provides interaction for drug molecules in the interlayer space enlarged by intercalation.^16^ The mechanisms involved in the interaction of the drug molecule-MMT hybrid system are hydrogen bonding, ion exchange, electrostatic interactions, van der Waals interaction, and hydrophilic/hydrophobic interaction.^14,15,17–19,22,23^ Na-Mica-4 is a highly charged, expandable synthetic phyllosilicate, with a theoretical interlaminar load of +4 and the formula Na_4_[Si_4_Al_4_]­Mg_6_O_20_F_4_·nH_2_O, with no natural equivalent and with the ability to expand in contact with water, while its charge is in the range of brittle micas.^24^ The high charge density is the result of the isomorphic substitution of Al^3+^ for Si^4+^ in the tetrahedral sheet. These high-charge swelling micas are interesting nanoclays due to their ease of synthesis, low cost, controllable composition, purity, and unique combination of high cation exchange capacity and swelling behavior. The attributes of Na-Mica-4 are recognized for their efficacy as adsorbents or in ion-exchange reactions to eliminate residual drug molecules. Therefore, exploring its potential as a carrier for pharmaceutical products offers promising research prospects.^20,25–27^

A cation-exchange reaction between the Na^+^ ions housed in the interlaminar space to compensate for the excess negative charge of the sheets, and cationic alkylammonium surfactants, causes changes in the interlaminar thickness, modification of the surface electric charge, and hydrophilic character of Na-Mica-4, creating a hydrophobic material. The physicochemical properties of organoclays can be relevant in water treatment, waste disposal, and biomedical applications depending on the chemical structure of the compounds.^28,29^ Therefore, it is interesting to compare the retention effectiveness of Cur as a hydrophobic drug, with inorganic clays, and investigate its use as a support for drug administration.^20,30,31^

Of the three clay types studied in this work, both MMT and Na-Mica-4 are characterized by the presence of negatively charged sheets with interlayer cations, while in the case of C18-Mica-4, hydrophobic spaces are expected at the interface due to the presence of amines. Given the initially assumed neutral or anionic characteristics of Cur at the pH used in this study, it would be expected that the C18-Mica-4 case would be the most reasonable for the hydrophobic regions of the ammonium-treated clay to favor Cur adsorption. However, a comparative study of the three types of clay is necessary to verify whether this behavior is fulfilled once the clay complex is formed with Cur, which, in general, has proven not to be easy to adsorb on clays.

This work aimed to investigate the possibility of having Cur complexes with different types of clay minerals that could be used as a Cur support that would improve its stability properties.

## 2 Experimental Section

### 2.1 Materials and Reagents

MMT from Patagonian (Rio Negro, Argentina) supplied by Castiglioni Pes and Co. was used as received. The chemical composition was [(Si_3.83_Al_0.11_)­(Al_1.43_Fe^3+^_0.26_Mg_0.30_)­O_10_(OH)_2_] Na_0.30_Ca_0.09_K_0.01_, the mineral composition was Na-montmorillonite (>99%) with quartz and feldspar as minor phases,^32^ and the CEC was 82.5 mequiv/100 g of clay.^33^

SiO_2_ (CAS no. 112945-52-5, 99.8% purity), Al­(OH)_3_ (CAS no. 21645-51-2), MgF_2_ (CAS no. 7783-40-6), and NaCl (CAS no. 7647-14-5, ≥99.5% purity) used for Na-Mica-4 synthesis were purchased from Sigma-Aldrich (Madrid, Spain). Octadecylamine (CAS 124-30-1, ≥99.0% purity) used for the organic functionalization of Na-Mica-4 was also purchased from Sigma-Aldrich.

Curcumin (Cur, CAS no. 458-37-7) was purchased from Sigma-Aldrich (Barcelona, Spain). Hydrochloric acid (Panreac, CAS 231-595-7), sodium hydroxide (Panreac, CAS 1310-73-2), and hydrogen peroxide (QuimiPur S.L.U. CAS no. 7722-84-1) were of high analytical quality. Solvents were of HPLC quality.

### 2.2 Synthesis of Highly Charged Swelling Micas

Na-Mica-4 was synthesized by the NaCl melt method following a procedure similar to that described previously (Alba et al., 2006). Its structural formula is Na_4_[Si_4_Al_4_]­Mg_6_O_20_F_4_·nH_2_O. The cation exchange capacity (CEC) is 291 mequiv/100g, calculated following the methodology reported by Choo and Bai in 2016.^34^ The starting products used were SiO_2_, Al­(OH)_3_, MgF_2_, and NaCl. The reactants were weighed and mixed in an agate mortar until the mixture was homogeneous.

Heat treatments were carried out in a Pt crucible at 900 °C for 15 h at a heating rate of 10 °C·min^–1^. The product was washed with deionized water, and the solid was separated by filtration, dried at room temperature, and then ground in the agate mortar.

### 2.3 Organofunctionalization of Na-Mica-4

Organomica C18-Mica-Na was prepared by a cation-exchange reaction between mica and an excess of primary alkylamine octadecylamine (2 CEC of Na-Mica-4).^35^ The primary amines were dissolved in an equivalent amount of HCl (0.1 M), and the resulting mixture was stirred for 3 h at 80 °C. The octadecylamine (ODA) solution was then mixed with 0.6 g of Na-Mica-4 and stirred for 3 h at 80 °C. Hot deionized water was added, and the mixture was stirred for 30 min at 50 °C, and then, the dispersion was centrifuged in an Eppendorf 5430 R centrifuge at 7800 rpm for 30 min at 5 °C. The product was dissolved in a hot ethanol:water mixture (1:1), stirred for 1 h, and centrifuged at 8000 rpm for 30 min at 5 °C. Finally, the precipitate was allowed to dry at room temperature. With the process described, a quaternary ammonium cation was formed by the amine and HCl, which then ion exchanges with the interlayer cation.^20,28^

### 2.4 Adsorption Studies

Water/ethanol solutions (50% (w/w)) were prepared below the solubility of Cur in this medium (0.133 mg/mL). Samples were kept stirring for 24 h until complete dissolution using topaz glassware to protect Cur from factors such as temperature and light. Poststirring, filtration was performed using 0.45 μm filters (Millipore, Darmstadt, Germany). Additionally, the pH of these solutions was measured to monitor stability and complex formation.

The Cur–clay complexes were formed in 2:1 w/v ratios of clay and Cur solution, respectively. In general, in other works, a Cur:clay ratio of 1:1 was chosen.^15,23^ In this work, a 2:1 ratio was chosen, with excess Cur in comparison since it was considered that an excess contribution was preferable to not having enough Cur for possible adsorption. For complex formation, 150 mg of clay and 75 mL of the prefiltered Cur solution were used. The Cur solution and clay were brought into contact and continuously stirred for 24 h. MMT, Na-Mica-4 clays, and C18-Mica-4 clays were used for Cur adsorption. The samples were then centrifuged in an Eppendorf 5430 R centrifuge at 7,800 rpm and 4 °C for 15 min. The resulting supernatant was collected and underwent a second filtration using 0.45 μm filters. Unadsorbed Cur was determined through HPLC, while the residue was dried for further characterization measurements according to Section 2.5. Before quantification through HPLC, pH measurements were conducted to detect potential variations during the complex formation.

Quantification was performed by HPLC (Hitachi Elite LaChrom, San Jose, CA, USA). The analytical method was optimized using a column Agilent Zorbax SB C-18 (4.6 × 150 mm, 3.5 μm) by following a method previously proposed by Musfiroh et al.^36^ The mobile phase consisted of 2% acetonitrile:acetic acid (50:50 v/v). The flow rate was fixed at 1.2 mL/min, and the injection volume was 20 μL. The absorbance was measured at 420 nm. Under these conditions, the peak areas were measured, and the HPLC analysis was conducted at 25 °C.

### 2.5 Characterization Methods

X-ray diffraction (XRD) data were obtained in a Bruker D8 Advance A25 diffractometer (Bruker, Germany) in a Bragg–Brentano configuration. The detector was a Lynxeye PSD detector (Bruker, Germany) equipped with a copper Kα radiation source (λ = 0.15405 nm). Measurements were taken with a 2θ range between 1° and 70°, a step size of 0.03°, a time per step of 0.1 s, tube conditions of 40 kV, and a current of 30 mA. The diffractometer was mechanically calibrated according to the manufacturer’s specifications, and a corundum standard was used to check the resolution in a wide range of angles.

Fourier transform infrared (FTIR) spectrum was recorded in the range of 4000–400 cm^–1^; after 32 scans, with a resolution of 4 cm^–1^, a Tensor II spectrometer (Bruker, Germany) was used. Samples were previously prepared using the KBr pellet technique. The pellets were prepared by pressing a mixture of sample and dried KBr (at 8 tons cm^–2^ for 2 wt % sample concentration).

Thermal gravimetric analyses (TGA) were performed on a Q600 STD instrument (TA Instruments, USA). The samples were heated from 25 to 900 °C at a heating rate of 10 °C/min in a nitrogen atmosphere.

Thermal analysis was performed using a DSC-131 instrument (Setaram, France). Samples (∼7–10 mg) were sealed in aluminum pans and measured in the temperature range from 30 to 300 °C with a heating rate of 10 °C/min. Several samples (between three and four) were analyzed for each type to ensure reproducibility of the results. An empty, sealed pan was used as a reference. Data were corrected by subtracting a baseline measured with an empty pan under the same conditions and were normalized by the sample weight. The specific surface area and the pore characteristics were determined by the Brunauer–Emmett–Teller (BET) method using an ASAP 2420 analyzer (Micromeritics, USA) with N_2_ sorption (Brunauer et al., 1938).

The surface charge of the clays was determined by correlation spectroscopy from electrophoretic mobility (μ) measurements, using Zetasizer Nano-S equipment (Malvern Instruments, Malvern, UK). The results were expressed as a zeta potential (Z, mV) after conversion of μ to Z by the Smoluchowski equation: *Z* = μη/ε, where η is the viscosity and ε is the permittivity of the solution.^37^ Measurements were made at room temperature, and 200 μL of samples was diluted with purified water (1/20).

Morphological studies of the solid complexes were performed by using scanning electron microscopy (SEM). A Teneo FEI electron microscope was used for Cur and Cur-loaded Na-Mica-4 after being subjected to stress conditions. Samples were spread on a support and sputter-covered with gold/palladium (Au/Pd 10 nm) under vacuum evaporation (4.5 · 10^–4^ Pa). A secondary electron detector was used.

### 2.6 Effect of Light on the Color of Cur and Cur-Mica-4 Complex

The effect of light on the variation of color parameters of the Cur and the Cur–clay complex was evaluated for one year. The solids were kept exposed to natural light cycles during this period. The “Commission Internationale of l’Eclairage” (CIE) 1976 color space system was applied to evaluate the color of the solids.^38^

Image acquisition was carried out with DigiEye Imaging System equipment. This apparatus includes a lighting box designed to adequately illuminate samples for color measurement, and a digital camera was connected to a computer. The cabinet is equipped with two fluorescent tubes that emulate the standard D65 illuminator and offer stable lighting conditions. The lamps were turned on at least 10 min before use, according to the manufacturer’s instructions, to stabilize them.^39^ CIELAB parameters were calculated using the original software CromaLab.^38^ The calculated CIELAB parameters were L* (the correlate of luminosity, which ranges from 0, black, to 100, white) and two-color coordinates, a* (which takes positive values for reddish colors and negative values for greenish ones) and b* (positive for yellowish colors and negative for bluish ones). From the coordinates a* and b*, other color parameters are defined: the hue angle (*h*_ab_, the correlate of chromatic tone) and the chroma (C*_ab_, the correlate of saturation). The differences in colors between samples were calculated using Δ*E*∗ = [(Δ*L*∗)^2^ + (Δ*a*∗)^2^ + (Δ*b*∗)^2^] 12 equation.^40^ The absolute lightness, chroma, and hue difference were calculated by a pair of samples (ΔL*, ΔC*_ab_, Δh_ab_).^41^

### 2.7 Forced Degradation Studies

Forced degradation studies involve a systematic investigation aimed at assessing the stability and degradation pathways of drug substances under various stress conditions.^42,43^ In this study, specific conditions were selected, and the methodology typically employed in such studies was as follows:

A) Hydrolytic conditions: Hydrolysis, a common degradation reaction across a broad pH range, involves exposing the drug substance to acidic or basic conditions to induce primary degradation. Initially, a reference solution of Cur (2.5 mg/mL) in ethanol was prepared. Then, 0.5 mL of 0.1 N NaOH (or HCl) was added, followed by a reaction period. Subsequently, 0.5 mL of 0.1 N HCl (or NaOH) was added to neutralize the pH.
B) Oxidation conditions: Oxidative degradation using hydrogen peroxide is common in forced degradation studies. This process involves an electron transfer mechanism to form reactive anions and cations. A 3% w/v hydrogen peroxide solution (1 mL) was added to 1 mL of a Cur solution (2.5 mg/mL) in ethanol, and the reaction was allowed to proceed for 8 h.
C) Photolytic conditions: Photostability testing assesses whether exposure to light leads to unacceptable changes in drug substances. Photostability studies involve exposing the substance to UV or sunlight to induce primary degradation. Starting with a standard Cur solution (2.5 mg/mL) in ethanol, an additional 15 mL of ethanol was added, and the solution was exposed to sunlight for 5 h. A similar procedure was conducted for UV exposure, with the duration extended to 8 h in a biosafety cabinet equipped with UV light at 254 nm.
D) Thermal conditions: Thermal degradation involves subjecting the standard Cur solution (2.5 mg/mL) diluted with 15 mL of ethanol to a thermostatic bath at 60 °C for 1 h.

Mica–Cur samples underwent the same procedure under all conditions, ensuring a 2.5 mg of Cur-loaded complex. Once each assay was completed, the samples were analyzed by HPLC following the chromatographic method described in the previous section. The characterization methods of Section 2.5 were applied to all treated samples following the solid preparation procedure explained in Section 2.4.

## 3 Results and Discussion

### 3.1 Adsorption Studies

Table 1 compiles the results on the concentration of Cur in the supernatant medium after its complexation with the clays studied (MMT, Na-Mica-4, and C18-Mica-4), along with the percentage of adsorption and pH after the adsorption process.

**Table 1:** Cur Concentration (mg/mL), Percentage of Cur Adsorbed into the Clay, and pH of Dissolution after the Adsorption Process^a^

	Cur concentration (mg/mL)	Cur–clay adsorption (%)	pH
Cur-Mica-4	0.053 ± 0.003	60.15 ± 7.42	8.9
Cur-MMT	0.125 ± 0.033	5.64 ± 0.81	8.4
Cur-C18-Mica-4	0.133 ± 0.042	0	7.9

a Cur concentration before the adsorption process was 0.133 mg/mL, and the pH of Cur solution was 7.6.

Among the strategies to enhance the stability of Cur, the use of clay minerals has been proposed in this work. MMT, Na-Mica-4, and C18-Mica-4 were selected for the study based on the wide experience of the research group in these clays for other applications.^20,28,44,45^

After the different clays were in contact with Cur, the characterization of the samples showed that some interaction had occurred, as can be seen in Section 3.2 for the case of Na-Mica-4. The cur remaining in the supernatant solvent was higher in C18-Mica-4 > MMT > Na-Mica-4, resulting in higher adsorption percentages in the case of Na-Mica-4 (60.15%).

The presence of cations in unmodified mica could favor the retention of Cur at acidic pH by cation exchange, although the mechanism by which this would occur cannot be determined from the results obtained. Furthermore, protons in the medium make the equilibrium shift to the enol tautomeric form of Cur (Figure 1), leading to an increase in pH (8.9). Kazakova et al. (2022) investigated the keto–enol tautomerism of Cur in water–ethanol solutions with fumed silica, indicating that the structural features of Cur tautomers, in both individual and aggregated states, greatly influence their adsorption properties.^46^ On the other hand, comparing the adsorbed amount of Cur on Na-Mica-4 (Table 1) with its surface area, a multilayer adsorption in small regions of Cur could be the adsorption mechanism.

Concerning the organomica, the steric hindrance between the Cur structure and ODA on the C18-Mica-4 surface makes the interaction between both molecules difficult, and therefore, slight differences in pH values were obtained. In this regard, Benítez et al. demonstrated how the steric hindrance arising from the densely packed arrangement of ODA molecules on mica obstructed carbamation when CO_2_ molecules attempted penetration.^47^

Based on these results, Na-Mica-4 was selected to complete the formation and stability studies of the Cur clay complex.

### 3.2 Solid-State Characterization

#### 3.2.1 X-ray Diffraction

Characterization by XRD showed a slight variation in the Na-Mica-4 diffraction of the 001 plane before and after the adsorption of Cur, indicating a possible adsorption in the interlaminar space of the clay (Figure 2a). It is also shown in the XRD result for pure Cur for comparison (Figure 2b). No signal of Cur is observed in the XRD result of the complex. The experimental diffractogram obtained (blue line) and Le Bail^48^ best fit (red) for Na-Mica-4 before and after adsorption are shown in Figure 2c,d. The bottom gray line shows the deviation between the best fit and experimental diffractograms.

**Figure 2:**
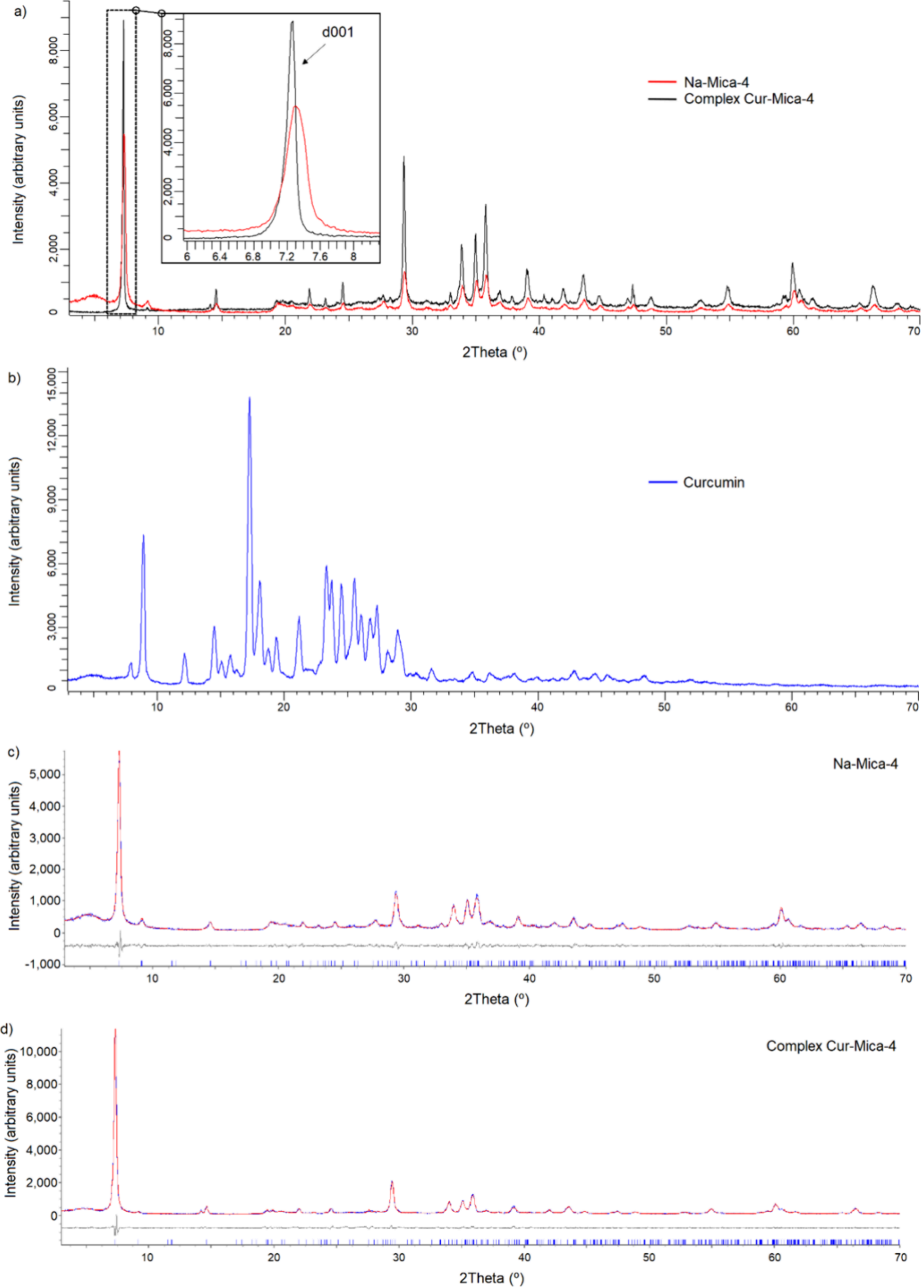
(a) Experimental diffractograms obtained for Na-Mica-4 (red) and Na-Mica-4 after the Cur adsorption assay (black) and enlargement of the region of reflections 001. (b) Diffractogram of Cur (blue) for comparison. (c,d) Experimental diffractogram obtained (blue line) and Le Bail best fit (red) for Na-Mica-4 (c) and Na-Mica-4 after the Cur adsorption assay (d). The bottom gray line shows the deviation between the best fit and experimental diffractograms.

Le Bail^48^ fittings were conducted using TOPAS 6 software^49^ and the fundamental parameter method. It is a profile fitting technique used to extract precise information about the position, intensity, width, and shape of each individual peak in a diffraction pattern.^20,28^ The background was fitted by using a fourth-order Chebyshev polynomial. The values of the goodness of fit (GOF) of the adjustments were checked to obtain values close to unity. At the same time, residual factor values (Rwp and Rbragg) were obtained. In the reported results, these values were generally small, indicating coherent data.^50^

GOF value obtained from the Le Bail fitting for Na-Mica-4 was 1.72, and Rwp and Rbragg were 10.72 and 0.557, respectively. The structure used was monoclinic in the space group P21, and the lattice parameters were as follows: *a* = 9.6833(15) Å, *b* = 5.1888(8) Å, *c* = 12.164(2) Å, β = 91.642(7)°, and *d* = 11.90 Å and 2θ = 7.43° for the (001) plane. After the adsorption process of Cur, the GOF, Rwp, and Rbragg were 2.81, 14.07, and 0.706, respectively. The lattice parameters were as follows: *a* = 9.6208(15) Å, *b* = 5.2288(8) Å, *c* = 11.9918(19) Å, β = 90.987(6)°, and *d* = 11.99 Å and 2θ = 7.37° for the (001) plane. The results obtained showed similar values for d in both cases, with a slightly higher value for the complex (Figure 2a). However, the difference in *d* between the mica and the complex is only 0.09 Å, which is a very small value compared to the size of Cur itself; hence, it cannot really be concluded that Cur is adsorbed at the Mica interface and might be adsorbed on the surface. The percentage of crystallinity was estimated by using Diffrac.EVA 5.2 software (Bruker). In the diffractogram of Cur (Figure 2b), higher crystallinity (74.0%) was obtained, and the phase was identified with the pattern PDF 00-066-1420 of Cur. The XRD results show a change in crystallinity after Cur incorporation, obtaining a value of 68.5% for Na-Mica-4 and 65.7% for the complex after the adsorption of Cur, so that the complex has a crystallinity lower than that of Na-Mica-4. The figure shows lower and wider peaks for the pure Na-Mica-4 case than for the complex, which is interpreted based on the size of the crystalline domains, having a smaller size in general for the pure Na-Mica-4 case (Figure 2a).

#### 3.2.2 Zeta Potential

The external surface charge of Na-Mica-4 was studied before and after the adsorption assays at pH ∼ 7. The Z potential resultant was −34.9 ± 4.2 and −13.9 ± 3.4 mV, respectively, suggesting a possible electrostatic interaction between the Cur and the clay surface. Since adsorption at the interface has not been observed by XRD, the lower value once Cur is adsorbed could also be explained by a lower surface charge density, since the Cur molecule has a relatively large size.

#### 3.2.3 BET Specific Surface

The results of the BET specific surface analysis^51^ for Na-Mica-4 before and after Cur adsorption were 3.00 ± 0.06 and 3.42 ± 0.05 m^2^/g, respectively; both showed a similar specific surface area.

However, the reduced pore size after the adsorption process (17.87 nm vs 13.12 nm before and after, respectively) was evidenced. The lower value in the pore size may be due to the immobilization of Cur molecules on the mica surface and the complex. As a result, it blocks the passage of N_2_ molecules, leading to clogging of pores in the structure.^52^

#### 3.2.4 Thermal Analysis

##### 3.2.4.1 Differential Scanning Calorimetry (DSC)

Differential scanning calorimetry (DSC) was employed to investigate the crystalline nature and interaction of Cur with Na-Mica-4. The results are displayed in Figure 3.

**Figure 3:**
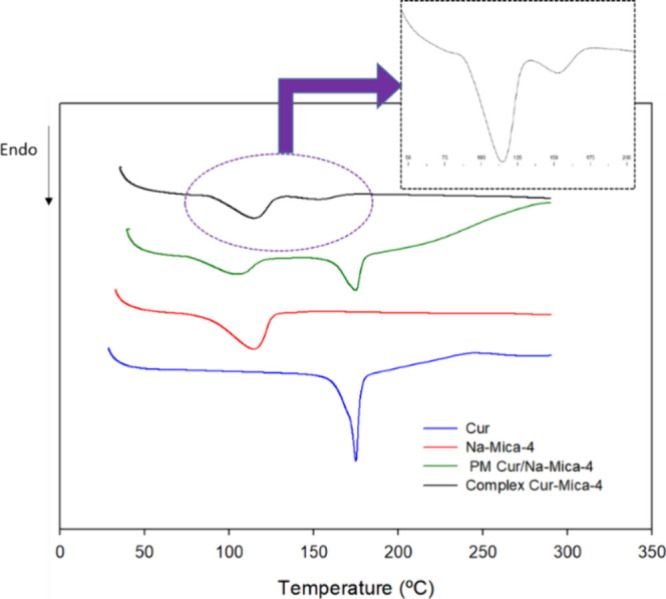
DSC thermograms of curcumin (Cur), Na-Mica-4, of the physical mixture 1:1 Cur and Na-Mica-4 (PM) and Na-Mica-4 (complex Cur-Mica-4). Heating rate: 10 °C/min.

The DSC curve of Cur is characterized by an endothermic sharp peak at 175 °C, in agreement with previously reported data.^14^ This prominent endothermic peak corresponding to the melting temperature of Cur indicates the crystalline nature of the drug. Na-Mica-4 showed a peak at 120 °C, resulting in water loss from the clay. This last peak is common in the 1:1 w/w physical mixture (PM) Cur + Na-Mica-4 and in the complex Cur-Mica-4.

Although the peak of Cur appeared in PM, the DSC data for the Cur-loaded mica complex displayed a peak that shifted relative to pure Cur. This shift indicates that Cur may interact with the clay matrix and undergo crystallization within the clay structure, resulting in melting at different temperatures. Similar findings were reported by Ahali Abadeh et al.,^53^ who evaluated the thermal behavior of Cur-loaded zeolite 5A samples using DSC. They demonstrated that the DSC peaks of Cur-loaded zeolites were shifted, suggesting that Cur crystallizes within the pores of the zeolite.

This leads to a lower melting point compared with free Cur. The peak shape differs, indicating the interaction with zeolite pores.^53^ These results complement XRD data, suggesting a change in Cur crystallinity postincorporation. This fact has also been described in the literature by other authors for other drugs. For example, DSC studies carried out by Makwana et al. revealed a change in the crystallinity of efavirenz when incorporated into solid lipid nanoparticles.^54^

Moreover, the shape of the melting peak differs: while free Cur exhibits a sharp peak, drug melting in combination with mica results in a broader peak. This distinction is attributed to the surface chemistry of mica and the specific interaction between the drug and this clay.^53^

##### 3.2.4.2 Thermal Gravimetric Analysis (TGA)

Thermogravimetric measurements were performed for the Cur–clay samples to determine the thermal stability and evaluate the drug load in the clay. Na-Mica-4 and Cur-loaded mica were examined by TGA, and the results of weight loss (%) and its derivative (%/°C) are shown in the Supporting Information (Figure S1) (Supporting Information is available in the online version of this work).

Reviewing the literature, Cur could remain stable up to 170 °C, a maximum loss of the material at 390 °C, followed by complete degradation at about 590 °C due to the boiling point.^14,55^

The Na-Mica-4 TG curve and derivative (DTG) presented three main mass loss events with percentages of mass loss equal to 0.89, 0.16, and 1.35% at the ranges <100, 100–200, and 500–900 °C, respectively. Initially, a weight loss of 0.89% occurred at a temperature lower than 100 °C due to the evaporation of water retained in the surface and interlayer space. This causes a decrease in the heat flow corresponding to an endothermic peak, as heat is required in the process, as shown in Figure 3. A weight loss of 0.16% occurred between 100 and 200 °C mainly due to the loss of interstitial water and slightly to the degradative effect of temperature on Cur; last, the weight loss of 1.35% in the range 500 and 900 °C is primarily due to clay mineral dihydroxylation, as was reported by Orta et al.^20^ Figure 4 illustrates the TG curves for Na-Mica-4 before and after Cur incorporation. Comparing the TG curve of Na-Mica-4 and Cur-loaded mica, a higher weight loss was obtained at temperatures higher than 500 °C (0.87%), attributable to the decomposition of the organic material present in the Cur-Mica-4 sample. Martin et al. obtained similar results when contaminants from water samples were adsorbed in C18-Mica.^28^

**Figure 4:**
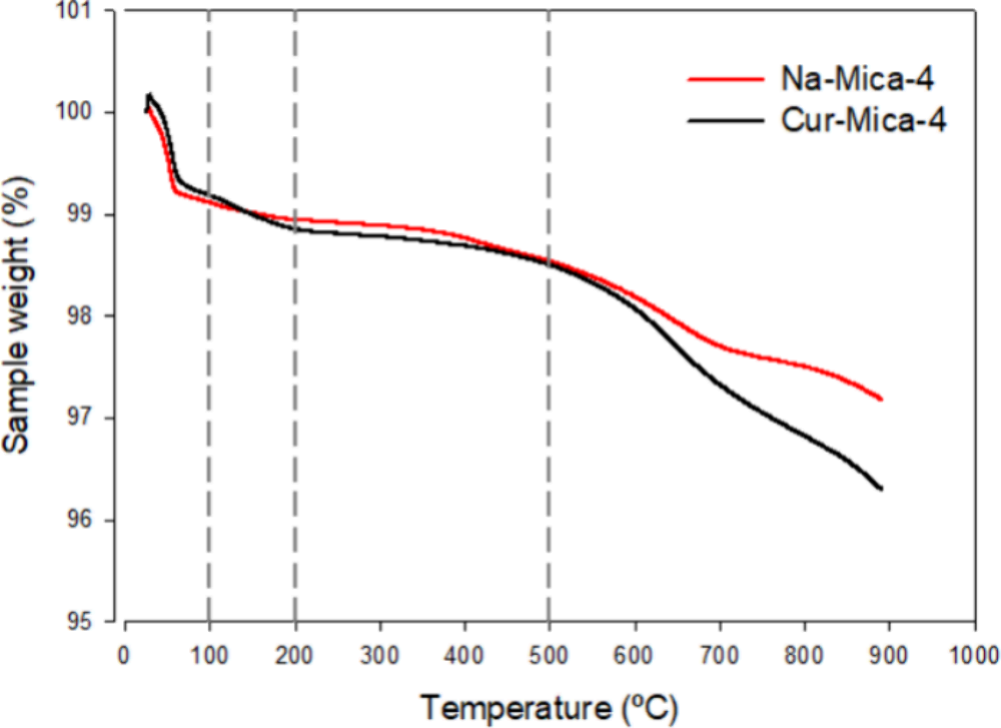
TG curves of curcumin-loaded Na-Mica-4 (Cur-Mica-4) and Na-Mica-4.

#### 3.2.5 Fourier Transform Infrared (FTIR) Spectroscopy

The FTIR spectrometry results demonstrate the formation of the Cur-Mica-4 complex (Figure 5), the detection of the characteristic bands of the Cur demonstrates the formation of complex, and they would not be detectable with the intensity shown if there were only a mixture of the compounds that form the complex as the quantitative proportion of Na-Mica-4 with respect to Cur (ratio 2:1 clay/solution of Cur 0.133 mg/mL, see Section 2.4). The bands at 965 and 452 cm^–1^ correspond to the Si–O–Si and Si–O–Al groups of the silicate layers, which shift to higher frequency in Na-Mica-4.^56^ The Cur-Mica-4 spectrum shows the following bands corresponding to the structure of Cur,^57^ stretching vibrations at 1628 cm^–1^ attributed predominantly to overlapping stretching vibrations of alkene (C=C) and carbonyl (C=O) characteristics. At 3200–3500 and 1427 cm^–1^, the stretching vibration attributed to the O–H and aromatic C=C groups is observed, respectively. A high intensity band at 1512 cm^–1^ is attributed to mixed vibrations of the stretching of the carbonyl bond, ν­(C=O), in-plane bending vibrations around aliphatic δ CC–C, δ CC=O, and planar bending around δ CC–H of the aromatic groups of Cur. The characteristic signals can be seen in the 2850–2960 cm^–1^ range of stretching vibrations of the C–H bonds related to the methoxy groups. A significant intense band at 1277 cm^–1^ is attributed to the bending vibration of the phenolic band ν­(C–O).^58^ The characteristic signals of the diketo form in the area between 1700 and 1750 cm^–1^ have not been detected, indicating that the Cur-Mica-4 complex is formed by Cur in formal enol, which is more stable than the diketo. Furthermore, a signal is seen in the absorption zone close to 550 cm^–1^, which is typical of the enol form of Cur.^59^

**Figure 5:**
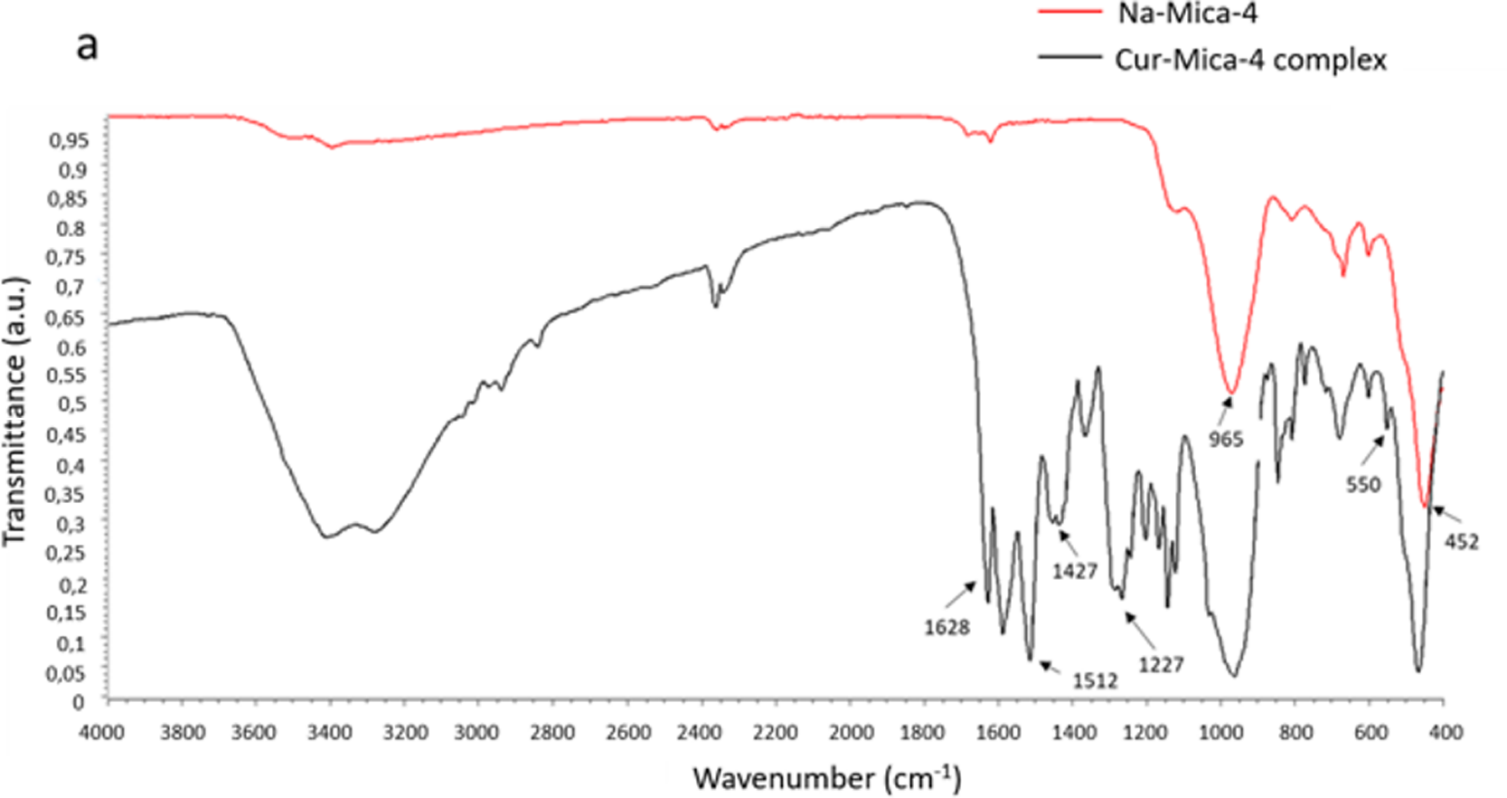
(a) FTIR spectra of Na-Mica-4 (red) and Na-Mica-4 after the adsorption assay of Cur (black).

### 3.3 Effect of Light on the Color of Cur and Cur–Clay Complex

The possible protection of Cur from exposure to natural light for one year was evaluated by calculating CIELAB parameters.

Table S1 shows the mean of the parameter values CIELAB (L*, a*, b*, *h*_ab_, and C*_ab_) for each measurement calculated during the overall study period. A higher luminosity and hue angle, a lower value of the colorimetric coordinates *a* and *b*, and the chroma value are observed in the Cur-Mica-4 complex than in Cur due to the colorimetric characteristics of the white clay. Table S2 shows the absolute lightness (ΔL*), chroma (ΔC*_ab_), and hue (Δ*h*_ab_) difference by a pair of samples (Tables S1 and S2 can be found in Supporting Information online).

The absolute color change, lightness, chroma, and hue difference (ΔE*, ΔL*, ΔC*_ab_, Δ*h*_ab_) for Cur and Cur-Mica-4 complex during the study period are shown in Table 2.

**Table 2:** Absolute Color Change (ΔE*), Lightness (ΔL*), Chroma (ΔC*_ab_), and Hue (Δ*h*_ab_) Difference of Cur and Cur–Clay Complex during the Study Period

	ΔE*	ΔL*	ΔC*_ab_	Δ*h*_ab_
Cur	5.83	1.55	–5.16	1.70
Cur-Mica-4 complex	12.56	0.94	–12.44	–1.35

The Δ*E* during the light exposure period of Cur is very subtle, indicating color stability of Cur against natural light exposure. The Cur–clay complex shows a somewhat greater variation; however, it cannot be considered significant, as this increase occurs mainly in the first 5 days of exposure.

### 3.4 Stability Studies of Cur-Loaded Na-Mica-4

The stability of Cur-loaded Na-Mica-4 has been studied under different environmental and working conditions: acidic, alkaline, and oxidizing media, as well as sunlight, UV, and thermal exposure. The morphological and structural characterization techniques TGA, DRX, SEM, and BET were used to draw conclusions about the behavior of clay as a protective structure.

#### 3.4.1 Morphology

The morphological structures of Cur-Mica-4 complexes exposed to stress conditions were examined by SEM analysis (Figure 6). Cur, Na-Mica-4, and Cur-Mica-4 samples in the absence of this treatment were included as references.

**Figure 6:**
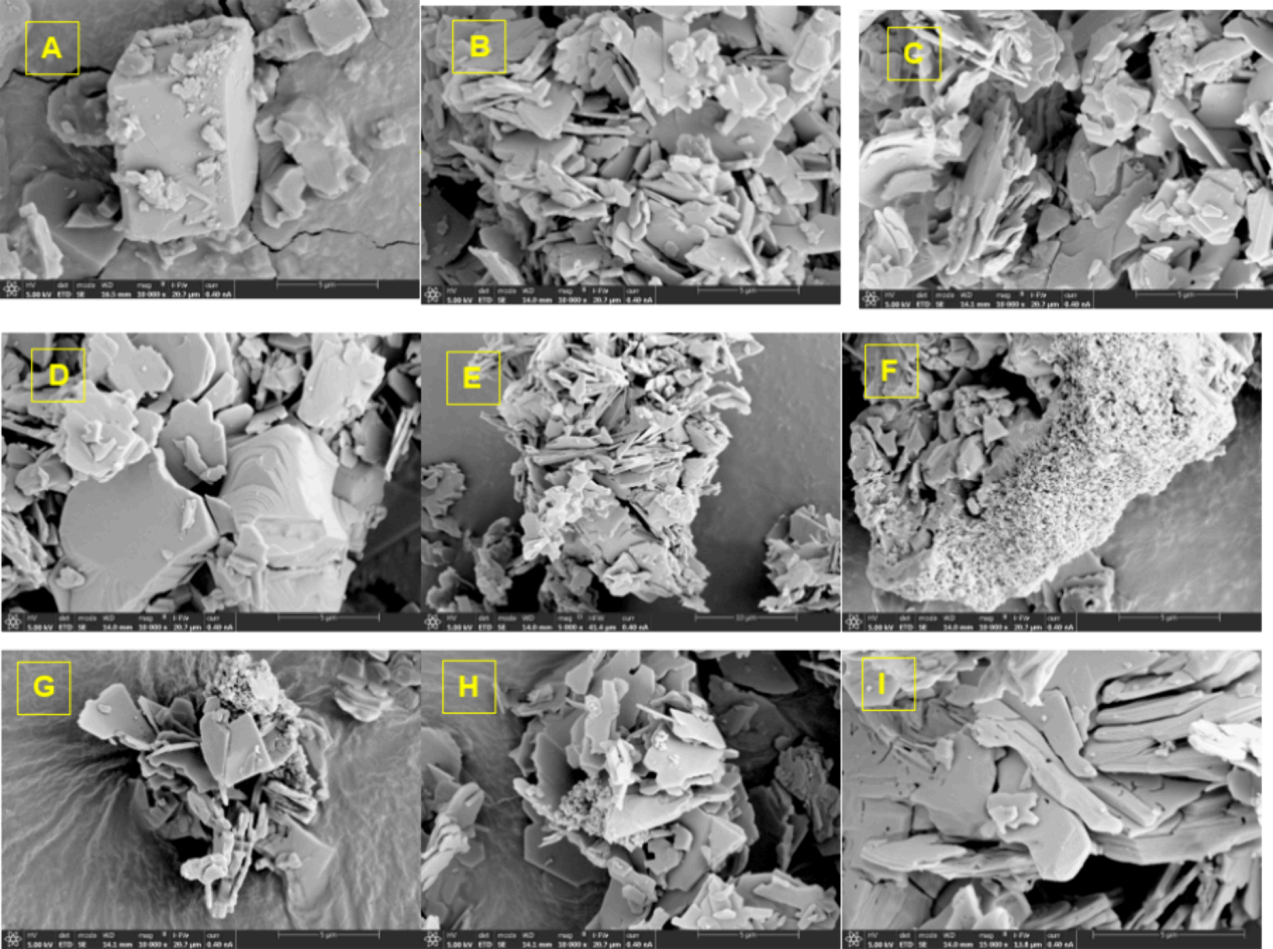
SEM images of (A) curcumin; (B) Na-Mica-4; (C) Cur-Mica-4; (D) Cur-Mica-4 after acid conditions; (E) Cur-Mica-4 after basic conditions; and (F) Cur-Mica-4 after peroxide conditions. (G) Cur-Mica-4 after sunlight conditions; (H) Cur-Mica-4 after heat conditions; and (I) Cur-Mica-4 after UV conditions.

According to the SEM images, differences in morphology were found between the different samples after exposure to stress conditions. Samples subjected to acidic conditions show large-sized structures, which has been related in previous works with expanded interlamellar spaces,^60^ as observed in Figure 6D. This conformation would facilitate the entry of Cur into clay, potentially lodging in the interlamellar spaces more effectively than elsewhere. This result was supported by Komadel and Madejová, which confirms the activation of clay in acidic media.^60^ Under alkaline conditions (Figure 6E), the structure of the mica reorganizes itself, forming irregular aggregates but maintaining, a priori, the structure of the complex before being subjected to the basic medium (Figure 6C). A new configuration emerges in the Cur-Mica-4 samples when exposed to oxidizing agents (H_2_O_2_), sunlight, and heat phenomena. In all cases, there is the appearance of aggregates, resembling a ‘sponge’ form, which do not correspond to Cur (Figure 6A), mainly due to its size and morphology, but maybe due to alterations of the clay itself under these conditions, as supported by the results obtained by López-Serrano et al. after treating different clays in alkaline hydrothermal waters. In their work, numerous spherical aggregates of sodalite displayed a cauliflower-shaped morphology on the material surface.^61^ Finally, UV radiation appears to be the phenomenon that would most significantly alter the morphology of mica; larger plate-like particles with large holes in darker areas of the image appear in its structure. Perforations could affect the stability of both the clay and Cur included within it. This strategy has been used by several authors using photocatalysis for wastewater remediation^62^ and as a support for removing pharmaceuticals from residual water.^63^

#### 3.4.2 Thermal Analysis

TG results are collected in Figures 7 and 8, and Table 3. TG and DTG of samples submitted to all forced degradation conditions are reported in the Supporting Information (Figure S1).

**Table 3:** Values of Mass Losses in Each Event Obtained from TG/DTG at the Following Temperature Ranges: (1) 30–100 °C, (2) 100–200 °C, and (3) 500–900 °C^a^

sample	WL1 (%)	WL2 (%)	WL3 (%)	WL3 (mg)	total WL (mg)
Na-Mica-4	0.89	0.16	1.35	0.14	0.26
Cur-Mica-4	0.94	0.35	2.22	0.27	0.43
acidic	6.48	2.90	4.37	0.28	0.89
alkaline	5.06	4.86	4.34	0.36	1.18
peroxides	5.13	1.64	2.24	0.21	0.83
sunlight	5.42	1.66	1.63	0.11	0.61
thermal	5.19	1.51	1.92	0.07	0.31
UV	5.42	1.51	1.86	0.12	0.57

a WL: Weight loss.

**Figure 7:**
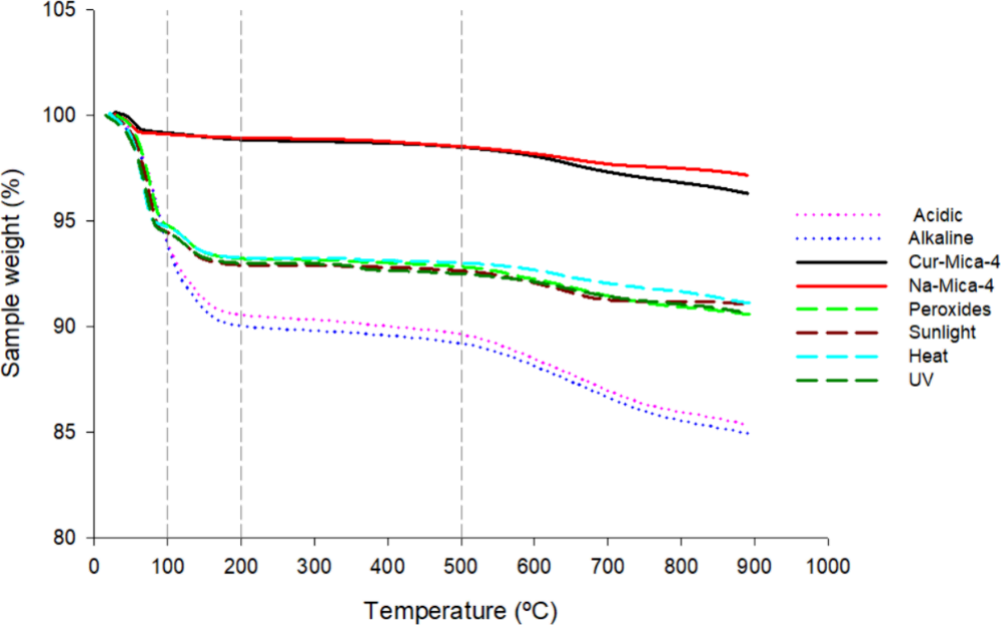
TG curves of Cur-Mica-4 after submitting to stress conditions. Na-Mica-4 and Cur-Mica-4 curves are plotted as references.

**Figure 8:**
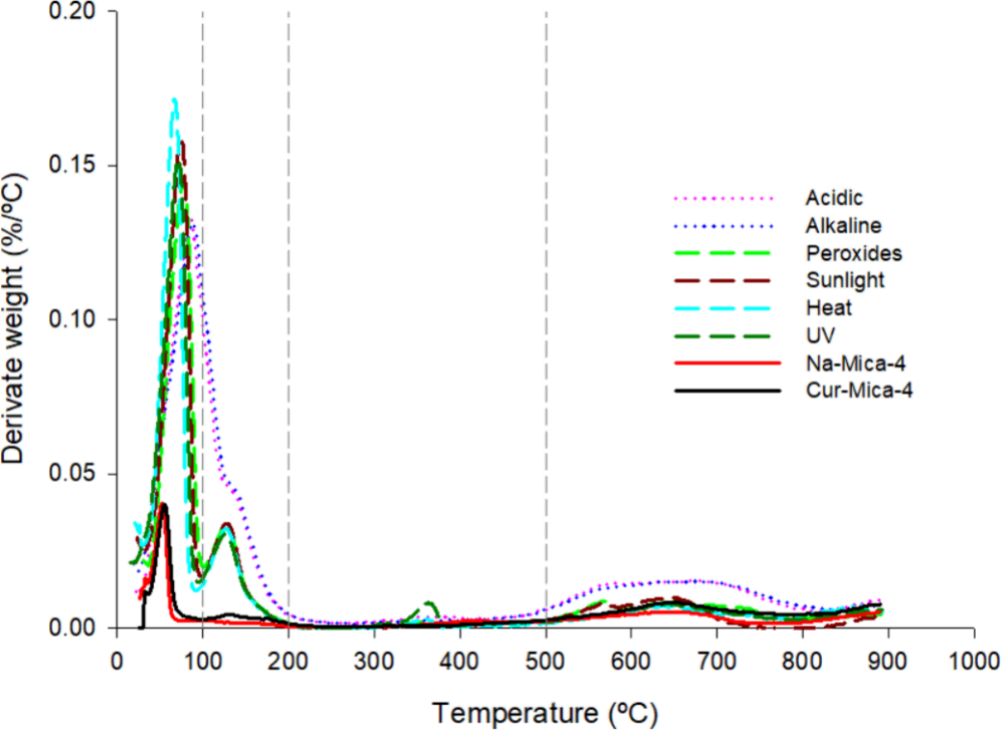
DTG curves of Cur-Mica-4 after exposure to stress conditions. Na-Mica-4 and Cur-Mica-4 curves are included as references. Solid line: Na-Mica-4 (red) and Cur-Mica-4 (black). Dashed line: Peroxides (light green), sunlight (brown), heat (blue), and UV (dark green). Dotted line: Acidic (pink) and alkaline (dark blue).

In the stress conditions tested, it was observed that all samples exhibited significant weight loss during the initial stage (30–100 °C) compared to Na-Mica-4 and Cur-Mica-4 samples, with the effect being more pronounced in acidic and alkaline environments (Table 3). Similar weight loss patterns within the temperature range of 30–100 °C have been frequently observed in previous studies. For example, Zhang and Wang reported comparable findings in their investigation of studying the thermal behaviors of polyacrylamide/clay composites based on various clays.^64^ This weight loss was attributed to the evaporation of water present in the interlayer space of the mica.

Between 100 and 200 °C, along with the substantial weight loss observed in samples exposed to acidic and alkaline conditions, a notable weight loss was also detected in samples subjected to peroxides, sunlight, heat, and UV, as indicated by the distinct peak in the DTG curve. Previous studies have attributed this weight loss in mica to the dehydration of interstitial water.^65^ Furthermore, comparing the Cur-Mica-4 complex before and after stress treatment, it is possible to speculate about a change in the layer conformation of mica and the overall structural configuration, potentially leading to enhanced water adsorption capacity within the pores.

In addition, samples exposed to acidic and alkaline environments exhibited higher weight loss in the final temperature range analyzed (500–900 °C), attributed to the decomposition of organic components, including Cur. Several studies confirm that the boiling temperature of Cur is 591.4 °C at 760 mmHg, which contributes to this degradative effect on weight loss.^66^

The total loss of matter (mg) in the analyzed samples (Table 3) demonstrates that those subjected to hydration generate a greater total mass loss than those samples, whose treatment did not require contact with an aqueous medium, precisely due to the adsorption of the aqueous medium in the interlayer and pores of the clay.^65^ Moreover, the results obtained from the mass loss in the last phase analyzed in the TGA (500–900 °C), where the decomposition of organic matter occurs, demonstrate the presence of Cur in the complexes after being treated with acidic, basic, and oxidizing agents, whose values resemble the complex before treatment (0.28, 0.36, and 0.21 mg, respectively, compared to 0.27 mg); in contrast, samples exposed to sunlight, heat, and UV radiation show residues similar to untreated mica (0.11, 0.069, and 0.12 mg, respectively, compared to 0.14 mg). This means that the factors that mainly contribute to the degradation of Cur and its disappearance from the sample during treatment are sunlight, heat, and UV radiation. On the other hand, acidic and basic conditions, while altering the structure of the mica, as can be seen in the microscopy study (Figure 6D,E), retain and would protect Cur through some bonds that would need to be elucidated in future studies.

TGA and SEM findings corroborate those of Gonçalves et al., who illustrated the improved stability of Cur through its interaction with clay minerals, particularly when compared to neutral and acidic environments. Additionally, their study also highlighted that the presence of clay mineral particles accelerated Cur photodegradation, attributed to the formation of oxygen products resulting from reactions between Cur and oxygen radicals.^3^ Schneider et al. provided results containing detailed information about the degradation of Cur, its products, and the autoxidative process in aqueous solutions. The formation of degradation products, such as a bicyclopentadione and other end products, in an autoxidative transformation, initiated by O_2_, results in the consumption of molecular oxygen,^67^ provided in this study by H_2_O_2_ (Figure 9).

**Figure 9:**
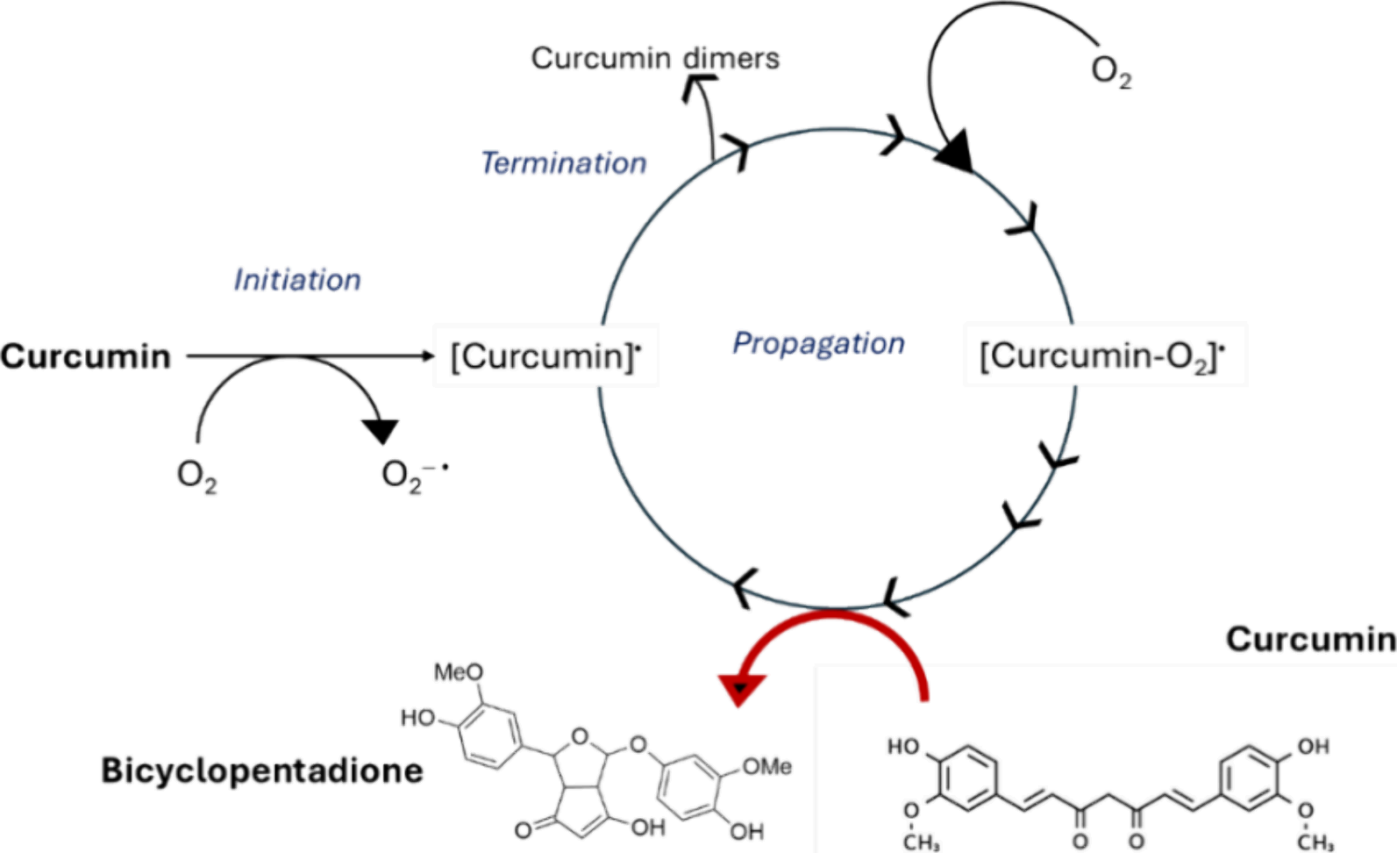
Autoxidation of curcumin in the presence of O_2_ to a bicyclopentadione.

#### 3.4.3 X-ray Diffraction

Table S3 of the Supporting Information shows the crystallographic parameters measured by the Le Bail method^48^ of the complexes before and after forced degradation tests, and Figure 10 shows the diffractograms obtained by XRD.

**Figure 10:**
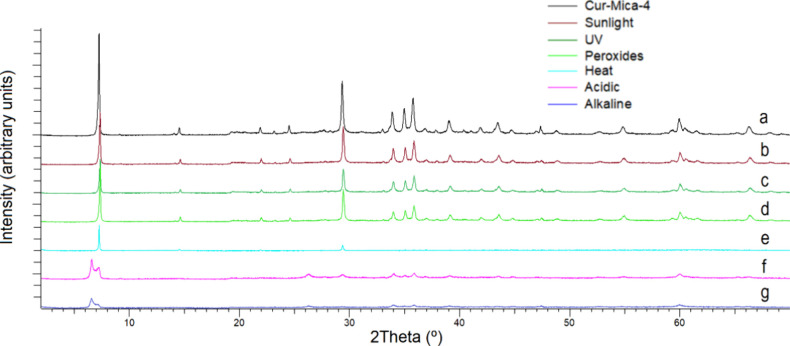
Diffractograms of the Cur-Mica-4 complex before forced degradation (a), after sunlight conditions (b), after exposure to UV light (c), after peroxide treatment (d), after heat conditions (e), after acid treatment (f), and after basic treatment (g).

The crystallographic structure of the micas after forced degradation tests maintains the same monoclinic structure as the original Na-Mica-4, although after the high-temperature treatment, the spatial group varies, remaining unchanged with the other degradative treatments. After the acidic and basic attacks of the complexes, double reflections at 001 are observed in the monoclinic structures.

#### 3.4.4 Specific Surface, Pore Size, and Pore Volume

Clay minerals contain a significant number of nanopores, which play a crucial role in providing space within shale gas reservoirs, particularly in reservoirs with low organic matter content during the low to mature stages.^68^

In this study, the mesopore parameters, including the specific surface (BET), average pore size, and total pore volume of eight samples obtained from N_2_ sorption, are listed in Table 4.

**Table 4:** Specific Surface Area, BET (m^2^/g), Total Pore Volume (cm^3^/g), and Pore Size (nm) of the Samples Evaluated

sample	BET (m^2^/g)	total pore volume (cm^3^/g)	mean pore size (nm)
Na-Mica-4	3.00 ± 0.06	0.0165–0.0171	27–40
Cur-Mica-4	3.42 ± 0.05	0.0128–0.0134	18–25
Acidic	2.18 ± 0.17	0.0176–0.0179	41–47
Basic	5.65 ± 0.06	0.0208–0.0211	18–19
Peroxide	4.17 ± 0.03	0.0164–0.0169	18–22
Sunlight	4.44 ± 0.03	0.0160–0.0163	18–21
Heat	3.12 ± 0.06	0.0158–0.0163	25–35
UV	3.11 ± 0.08	0.0119–0.0128	35–40

In the case of BET, the uncertainty is estimated from the regression residuals. For total pore volume and mean pore size, a range is established based on the difference in results from the BJH model between adsorption and desorption curves.

Typically, clays can alter their configuration when subjected to forced treatments such as exposure to acidic, basic, oxidizing conditions, sunlight, UV, or heat. This alteration affects the specific surface area of the sample, which may result in either an increase or a decrease in its value.

In this study, it was observed that samples subjected to various stress conditions exhibited changes in their properties. For instance, the BET surface area increased from 2.18 m^2^/g under acidic treatment to a maximum of 5.65 m^2^/g under alkaline conditions. Furthermore, the mean mesopore size decreased from 41–47 to 18–19 nm in these samples.

It should be noted that the specific surface area of Cur-Mica-4 prior to treatment was 3.42 m^2^/g. However, with the addition of acid, the specific surface area decreased to 2.18 m^2^/g, resulting in a larger size of the complex configuration. This result may be attributed to the presence of higher BET surface areas of expandable phyllosilicate structure after acidic treatment, as observed in SEM studies and corroborated by other research aimed at obtaining activated clays.^13^

Conversely, at a basic pH, an inverse relationship was observed, leading to a higher surface area with smaller pore sizes. Upon reviewing the literature, it appears that under basic pH conditions, the clay surface may undergo the formation of novel configurational structures referred to as streaks. These streaks are characterized as irregular swelling structures with bulge-type shapes. Their emergence was linked to an increased uptake of water influenced by localized structural variations.^69^ These findings may be supported by SEM images and TG analysis, where higher water loss was obtained from 30 to 200 °C.

It is important to highlight that the pore size acquired similar values in all samples compared to the Cur-Mica-4 sample, suggesting, as previously explained, that the presence of Cur in the complex could be impeding the N_2_ penetration. However, in the case of heated Cur-Mica-4, there is indeed an expansion in the pore size, aligning closely with Na-Mica-4. This suggests Cur degradation under these conditions.

UV radiation and sunlight did not increase the pore size in Cur-Mica-4. This is notable because TGA analysis demonstrated that these two factors triggered the degradation of Cur, which should translate into a higher pore size. However, as this was not the case, the Cur degradation products could block N_2_ flow across Cur-Mica-4 pores, reducing its size. Some of these products, such as vanillin, ferulic acid, or ferulic aldehyde, contain phenolic moieties that can be further intercalated into the interlayer spaces, as other authors demonstrated.^70^

## 4 Conclusions

This work investigates the possibility of having curcumin–clay mineral complexes that would improve Cur (Curcumin) stability properties. Cur adsorption tests have been carried out in different clay minerals; the high-charge expandable synthetic mica, Na-Mica-4, has been the one with which the best results were obtained.

The characterization techniques employed have demonstrated the formation of complexes between Na-Mica-4 and the enol tautomer of Cur, suggesting possible adsorption mechanisms through electrostatic and chemical interactions.

The color stability of both Cur and the complex in the solid state was not significantly affected after exposure to natural light for one year.

Regarding the stability of the complex under forced degradation conditions, acidic and basic conditions were the least detrimental to the stability of Cur. At the same time, UV light appears to have been the factor that most affected the Cur stability, according to the results obtained from both TG and SEM analyses.

The results obtained support the potential use of Na-Mica-4 as a gastroresistant release system for Cur due to the interaction of Cur with Na-Mica-4 clay. Furthermore, Orta et al. published the results of gastrointestinal resistance of organofunctionalized Mica-4, whose laminar structure is the same as that of Na-Mica-4.^30^

The stability studies of Cur in the complexes with Na-Mica-4 will be completed in the next studies by HPLC, in which both the drug and its degradation products after forced degradation conditions will be analyzed. LC-MS and NMR studies will also be performed to characterize the mechanisms of protection of Cur by Na-Mica-4.

New adsorption studies of Cur on C18-Mica-4 and Na-Mica-4, varying the pH and adsorption conditions, will be done to better understand why the complex formation occurred in this study on Na-Mica-4 and not on C18-Mica-4.

## Supplementary Material



## Notes

Table of CIELAB parameter values (*L**, *a**, *b**, *h*_ab_, and *C**_ab_) for each of the measures calculated during the overall study period; table of absolute lightness (Δ*L**), chroma (Δ*C**_ab_), and hue (Δ*h*_ab_) difference by pair of samples; table of crystallographic parameters of Cur–mica momplex before and after forced degradation tests; and figures of TG/DTG curves of Na-Mica-4 and Cur-Mica-4 before exposing to stress conditions and after this (PDF)
