# Supplementary material for: Curcumin-Loaded High-Charge Swelling Synthetic Mica: Characterization Studies and Stability under Stress Conditions
Source: Langmuir. 2025 May 23;41(21):13407–20. doi: 10.1021/acs.langmuir.5c01163 (PMC12139042; doi:10.1021/acs.langmuir.5c01163)
Supplement: Supplementary file 1 [file la5c01163_si_001.pdf]

Curcumin-loaded high-charge swelling synthetic mica. Characterizacion studies and stability under stress conditions

Orta Cuevas, María del Mar [(1)*], Fernández Romero, Ana [(2)], Rabasco Álvarez, Antonio M.[(2)], Medina-Carrasco, Santiago [(3)*], González-Rodríguez, María Luisa [(2)]

* Corresponding authors

(1) Department of Analytical Chemistry, Faculty of Pharmacy, Universidad de Sevilla, C/ Profesor García, González 2, 41012 Sevilla, Spain

(2) Department of Pharmacy and Pharmaceutical Technology, Faculty of Pharmacy, Universidad de Sevilla, C/ Profesor García, González 2, 41012 Sevilla, Spain

(3) X-ray Laboratory, CITIUS, Universidad de Sevilla, Avenida Reina Mercedes, 4B, 41012 Sevilla, Spain

Supplementary material

Table S1. CIELAB Parameter values (L*, a*, b*, h_{ab} and $C*_{ab}$) for each of the measures calculated during the overall study period

Date	Sample	L*	a*	b*	C*ab	hab
21/04/2022	Cur	66.18	40.19	71.56	82.10	60.63
	Cur-Clay complex	76.02	22.12	67.61	71.22	72.19
22/04/2022	Cur	69.71	40.78	72.39	83.10	60.56
	Cur-Clay complex	80.48	21.97	64.80	68.49	71.63
27/04/2022	Cur	69.54	40.69	71.74	82.50	60.40
	Cur-Clay complex	79.22	22.42	63.71	67.60	70.84
28/04/2022	Cur	66.20	39.59	73.00	83.07	61.42
	Cur-Clay complex	76.68	20.47	60.85	64.27	71.80
16/05/2022	Cur	65.33	38.99	69.36	79.59	60.62
	Cur-Clay complex	75.89	20.46	58.07	61.62	70.82
23/05/2022	Cur	66.18	38.79	72.02	81.83	61.60
	Cur-Clay complex	76.79	20.12	58.58	62.00	71.30
08/07/2022	Cur	65.86	38.59	69.15	79.21	60.82
	Cur-Clay complex	77.14	20.15	57.09	60.59	70.76
17/01/2023	Cur	65.60	38.16	67.65	77.69	60.58
	Cur-Clay complex	75.66	20.96	56.61	60.41	69.86
20/04/2023	Cur	67.73	35.77	68.08	76.94	62.33
	Cur-Clay complex	76.96	19.63	55.33	58.78	70.84

Table S2. Absolute lightness (ΔL^*), chroma (ΔC^*_{ab}) and hue (Δh_{ab}) difference by pair of samples.

	ΔE^*	ΔL^*	ΔC^*_{ab}	Δh_{ab}	Variation between measurements
**Cur**	3.67	3.52	1.00	-0.07	1st-2nd (21-04-22/22-04-22)
**Cur-Clay complex**	5.27	4.46	-2.72	-0.55	
**Cur**	0.67	-0.17	-0.60	-0.15	2nd-3rd (22-04-22/27-04-22)
**Cur-Clay complex**	1.73	-1.27	-0.89	-0.79	
**Cur**	3.73	-3.34	0.57	1.02	3rd-4th (27-04-22/28-04-22)
**Cur-Clay complex**	4.29	-2.53	-3.33	0.96	
**Cur**	3.78	-0.87	-3.48	-0.80	4th-5th (28-04-22/16-05-22)
**Cur-Clay complex**	2.89	-0.80	-2.65	-0.98	
**Cur**	2.79	0.85	2.24	0.98	5th-6th (16-05-22/23-05-22)
**Cur-Clay complex**	1.09	0.90	0.38	0.48	
**Cur**	2.89	-0.32	-2.63	-0.78	6th-7th (23-05-22/8-07-22)
**Cur-Clay complex**	1.53	0.35	-1.41	-0.54	
**Cur**	1.58	-0.26	-1.52	-0.23	7th-8th (8-07-22/17-01-23)
**Cur-Clay complex**	1.75	-1.48	-0.18	-0.90	
**Cur**	3.24	2.14	-0.75	1.75	8th-9th (17-01-23/20-04-23)
**Cur-Clay complex**	2.26	1.30	-1.64	0.98	

Table S3. Crystallographic parameters of Cur-Mica Complex before and after forced degradation tests (fdt)

Sample	Structure	Space group	Lattice parameters				d001(Å)	d001 (2Theta)
			a(Å)	b(Å)	c(Å)	beta (°)		
Cur-Mica complex	Monoclinic	P21	9.63(15)	5.2288(8)	11.9918(19)	90.987(6)	11.99	7.37
After fdt Acid	Monoclinic	P21	9.5949(18)	5.2016(10)	11.976(2)	91.826(12)	11.97	7.38
After fdt Alcaline	Monoclinic	P21	9.605(11)	5.219(7)	12.028(15)	92.12(2)	12.02	7.35
After fdt Peroxides	Monoclinic	P21	9.673(2)	5.2343(13)	12.043(3)	91.485(7)	12.04	7.34
After fdt Sunlight	Monoclinic	P21	9.675(2)	5.2350(14)	12.041(3)	91.475(7)	12.04	7.34
After fdt Thermal	Monoclinic	C2	5.321(3)	9.203(7)	12.191(8)	94.671(18)	12.15	7.27
After fdt UV	Monoclinic	P21	9.676(2)	5.2354(13)	12.044(3)	91.488(7)	12.04	7.34

Figure S1. TG/DTG curves of Na-Mica-4, Cur-Mica-4 before exposing to stress conditions and after this. A) Na-Mica-4. B) Cur-Mica-4. C) Acidic conditions. D) Alkaline conditions. E) Peroxides. F) Sunlight. G) Heat. H) UV radiation.

(A)

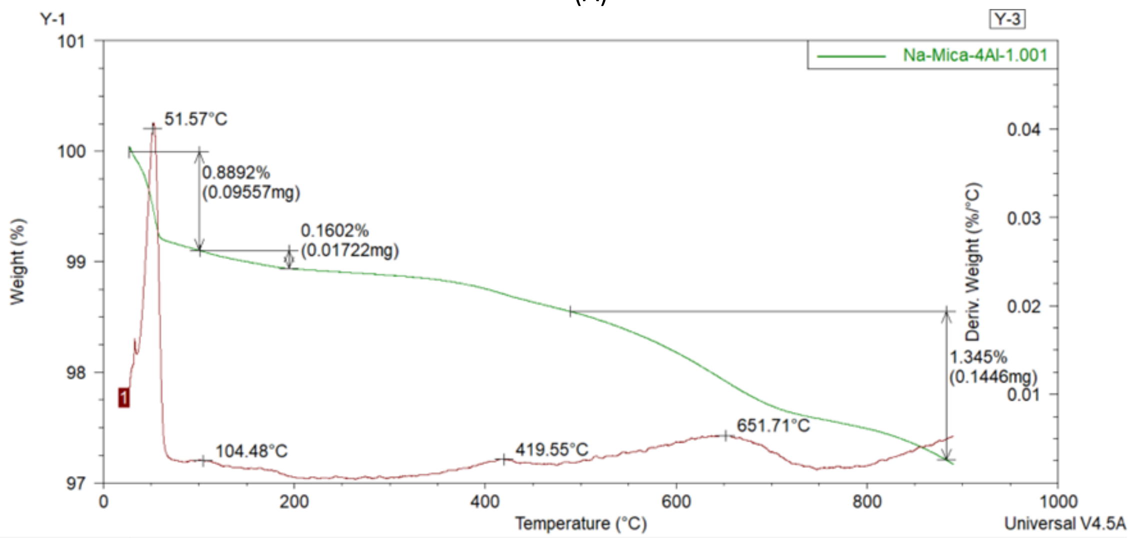

(B)

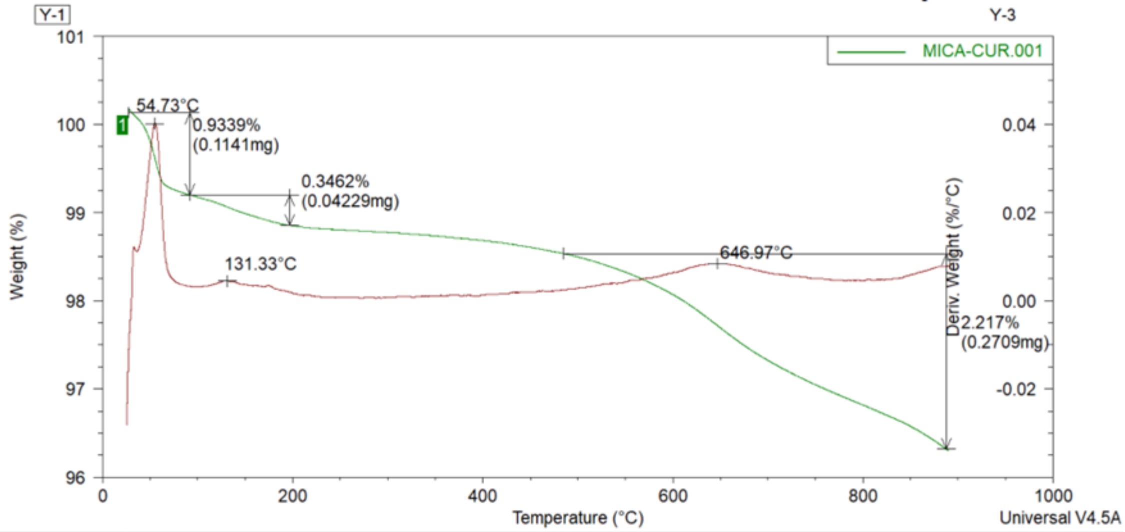

(C)

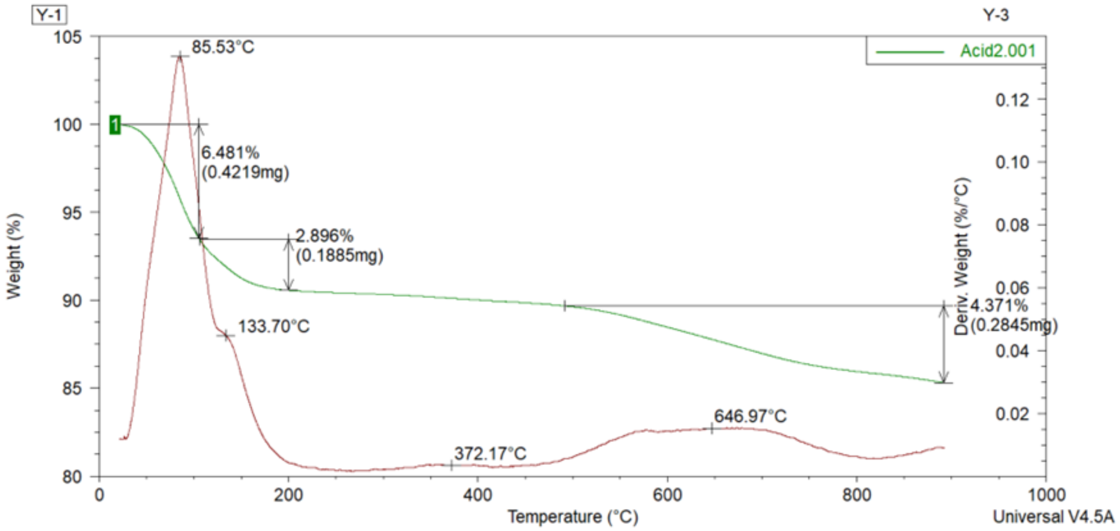
Y-1
Y-3
Acid2.001
85.53°C
6.481%
(0.4219mg)
2.896%
(0.1885mg)
133.70°C
4.371%
(0.2845mg)
646.97°C
372.17°C
Weight (%)
Deriv. Weight (%/°C)
Temperature (°C)
Universal V4.5A

(D)

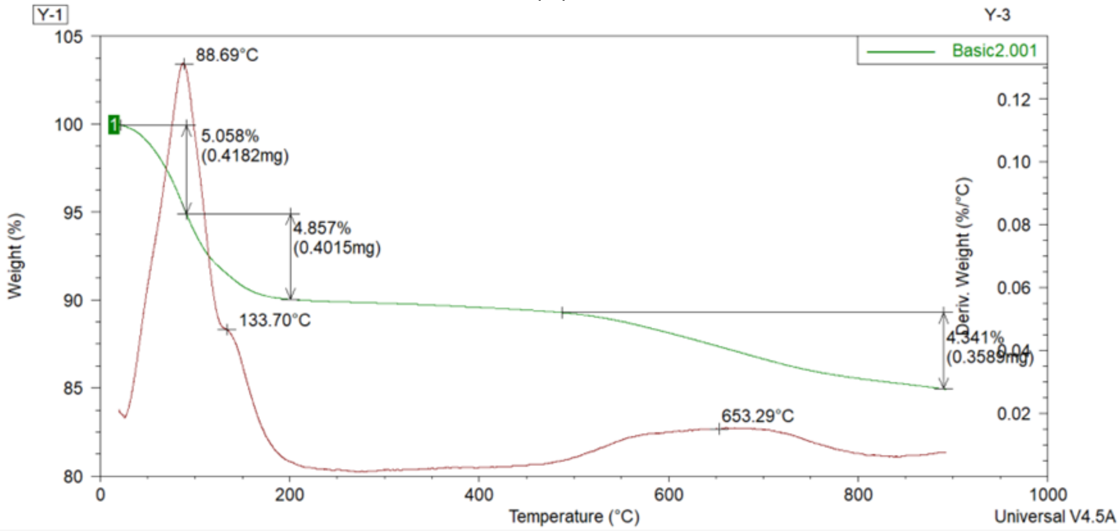
Y-1
Y-3
Basic2.001
88.69°C
5.058%
(0.4182mg)
4.857%
(0.4015mg)
133.70°C
4.341%
(0.3589mg)
653.29°C
Weight (%)
Deriv. Weight (%/°C)
Temperature (°C)
Universal V4.5A

(E)

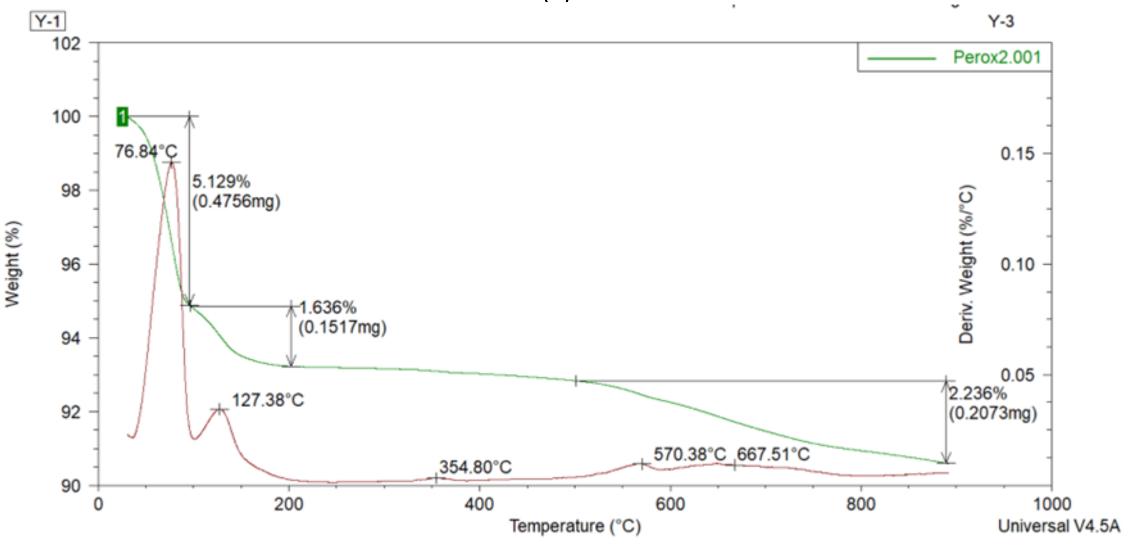
Y-1
Y-3
Perox2.001
76.84°C
5.129%
(0.4756mg)
1.636%
(0.1517mg)
127.38°C
2.236%
(0.2073mg)
354.80°C
570.38°C
667.51°C
Weight (%)
Deriv. Weight (%/°C)
Temperature (°C)
Universal V4.5A

(F)

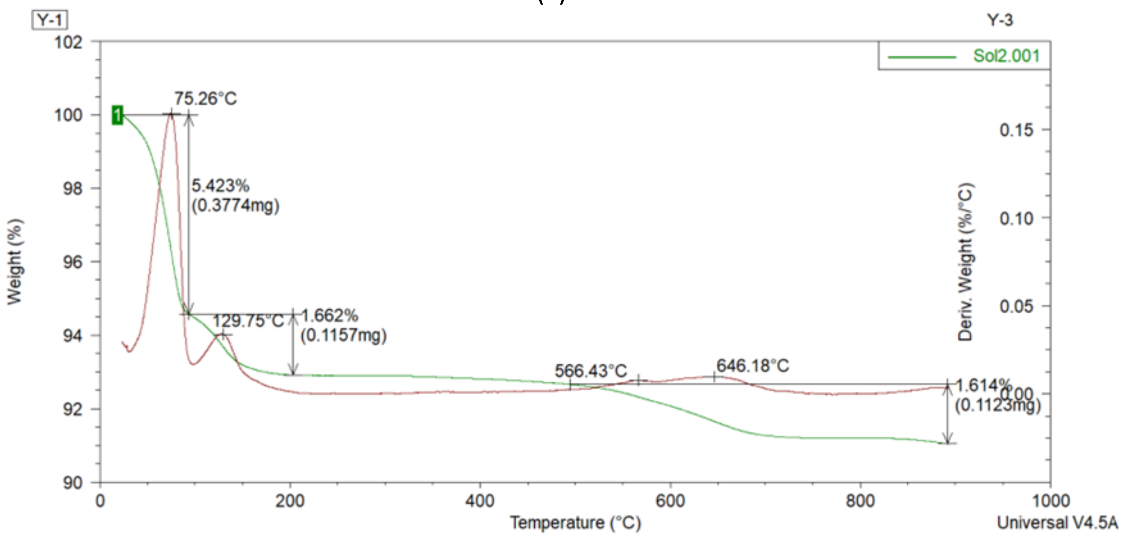
Y-1
Y-3
Sol2.001
75.26°C
5.423%
(0.3774mg)
129.75°C
1.662%
(0.1157mg)
566.43°C
646.18°C
1.614%
(0.1123mg)
Weight (%)
Deriv. Weight (%/°C)
Temperature (°C)
Universal V4.5A

(G)

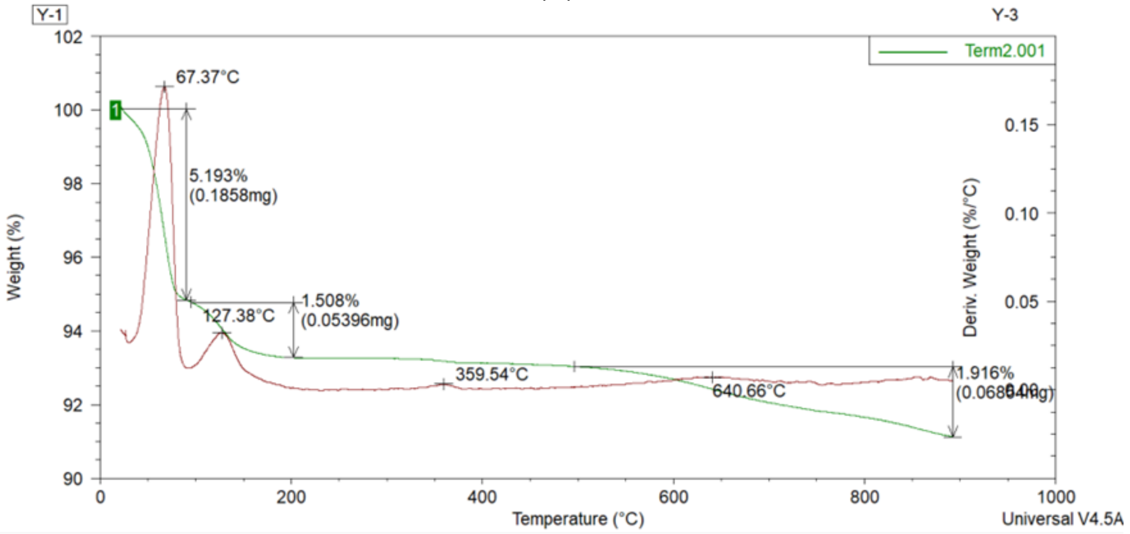
Y-1
Y-3
Term2.001
67.37°C
5.193%
(0.1858mg)
127.38°C
1.508%
(0.05396mg)
359.54°C
640.66°C
1.916%
Weight (%)
Deriv. Weight (%/°C)
Temperature (°C)
Universal V4.5A

(H)

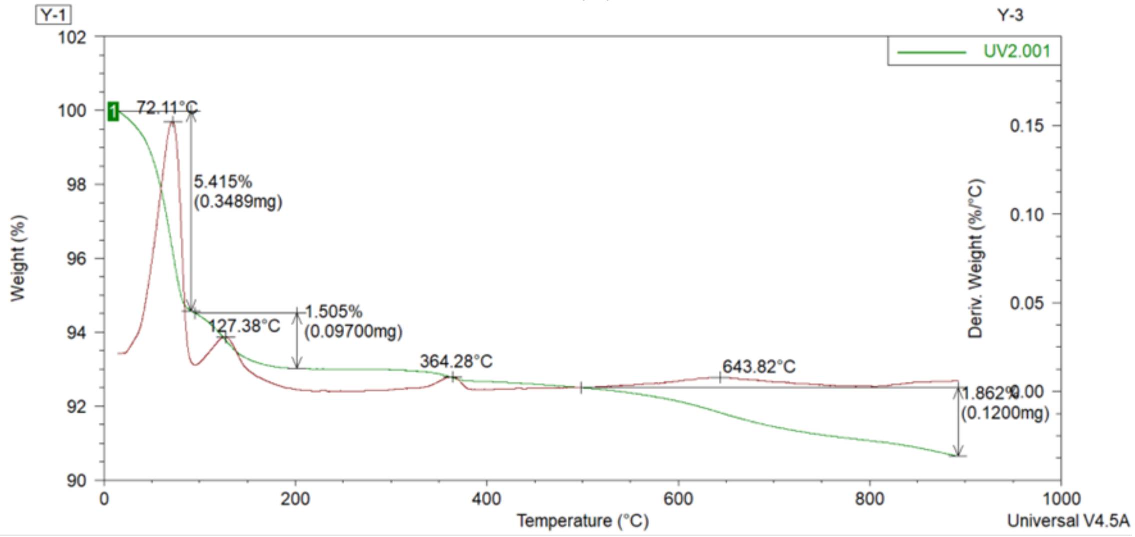
Y-1
Y-3
UV2.001
72.11°C
5.415%
(0.3489mg)
127.38°C
1.505%
(0.09700mg)
364.28°C
643.82°C
(0.1200mg)
Weight (%)
Deriv. Weight (%/°C)
Temperature (°C)
Universal V4.5A
